# Integrated bioinformatic analysis of RNA binding proteins in hepatocellular carcinoma

**DOI:** 10.18632/aging.202281

**Published:** 2020-12-19

**Authors:** Ling Wang, Zhen Zhang, Yuan Li, Yanyan Wan, Baocai Xing

**Affiliations:** 1 Key Laboratory of Carcinogenesis and Translational Research, Ministry of Education, Hepatopancreatobiliary Surgery Department I, Peking University Cancer Hospital and Institute, Beijing 100142, China; 2 Department of Gastroenterological Surgery, Peking University People’s Hospital, Beijing 100044, China; 3 Laboratory of Surgical Oncology, Beijing Key Laboratory of Colorectal Cancer Diagnosis and Treatment Research, Peking University People’s Hospital, Beijing 100044, China

**Keywords:** RNA binding protein, hepatocellular carcinoma, biomarker, transcriptomics, proteomics

## Abstract

RNA binding proteins (RBPs) are aberrantly expressed in a tissue-specific manner across many tumors. These proteins, which play a vital role in post-transcriptional gene regulation, are involved in RNA splicing, maturation, transport, stability, degradation, and translation. We set out to establish an accurate risk score model based on RBPs to estimate prognosis in hepatocellular carcinoma (HCC). RNA-sequencing data, proteomic data and corresponding clinical information were acquired from the Cancer Genome Atlas database and the Clinical Proteomic Tumor Analysis Consortium database respectively. We identified 406 differentially expressed RBPs between HCC tumor and normal tissues at the transcriptional and protein level. Overall, 11 RBPs (BRIX1, DYNC1H1, GTPBP4, PRKDC, RAN, RBM19, SF3B4, SMG5, SPATS2, TAF9, and THOC5) were selected to establish a risk score model. We divided HCC patients into low-risk and high-risk groups based on the median of risk score values. The survival analysis indicated that patients in the high-risk group had poorer overall survival compared to patients in the low-risk group. Our study demonstrated that 11 RBPs were associated with the overall survival of HCC patients. These RBPs may represent potential drug targets and can help optimize future clinical treatment.

## INTRODUCTION

Hepatocellular carcinoma (HCC) is the most common primary liver cancer. According to estimates from GLOBOCAN, HCC is the fourth leading cause of cancer death (8.2%) and ranks as the sixth most commonly diagnosed cancer (4.7%) worldwide in 2018 [1]. Currently, the optimal treatment for HCC patients is radical resection surgery at the early stage, but many cases are diagnosed at the advanced stage, leading to poor prognosis [2, 3]. With the development of high-throughput analyses of many HCC samples, researchers are starting to gain a deeper understanding of the molecular changes in cancer cells [4]. HCC cancer cells accumulate somatic DNA alterations, including mutations and chromosomal aberrations [5]. HCC patients at the same clinical stage tend to have different molecular subtypes, and evidence indicates that gene signatures have significant potential in predicting HCC patients’ prognoses [6]. Therefore, identification of novel biomarkers is necessary for early screening, diagnosis and molecular targets of HCC patients in order to improve survival rates.

RNA-binding proteins (RBPs) comprise a large family of proteins that binds RNA through RNA-binding domains (RBDs) and interact with the bound RNAs [7, 8]. RBPs bind to a variety of RNAs, including rRNAs, mRNAs, tRNAs, ncRNAs, snRNAs, and snoRNAs [9]. They play a vital role in post-transcriptional gene regulation (PTGR) and are involved in RNA splicing, maturation, transport, stability, degradation, and translation [9]. Mechanistically, the protein components of the ribonucleoprotein complexes (RNPs) with RBPs at their core participate in pre-mRNA processing and determine mRNA export, localization, translation and stability [10]. Additionally, RBPs and their interacting partners disrupt the core components of miRNA biogenesis and change the secondary structure of miRNA target sites, leading to dysregulation of miRNAs in specific tumor types [11]. A recent study has shown that the majority of RBPs are expressed at higher levels than the average cellular protein, and several RBPs are abnormally expressed during the development and progression of cancer [12]. Altered RNA metabolism due to malfunction of RBPs can cause intricate changes in the cellular transcriptome and proteome, leading to changes in cell proliferation, apoptosis, invasion and migration [13]. A growing number of studies have discovered that differentially expressed RBPs are associated with poorer prognoses in breast cancer and lung adenocarcinoma, suggesting that certain RBPs can act as promising targets for cancer therapy [12, 14–16]. Meanwhile, a new set of regulatory RBPs have been considered to play an important role in intestinal homeostasis, adaptation to injury, and participation in malignant transformation. The aberrant expression and function of these RBPs in colorectal cancer can help provide the impetus for developing inhibitors of these RBPs [17].

Although RBPs are known to be closely associated with the initiation and progression of various cancers, the comprehensive roles of RBPs in HCC remains unclear. The application of next-generation sequencing technology and modern protein mass spectrometry helps facilitate identification of changes in RBPs expression across HCC samples. In this study, we downloaded RNA-sequencing data from the Cancer Genome Atlas (TCGA) database and mass-spectrometry-based data from the Clinical Proteomic Tumor Analysis Consortium (CPTAC) database. After distinguishing consistently transcriptomic and proteomic alterations of RBPs between HCC tumor and normal tissues, we established a risk score model based on 11 prognostic RBPs. Ultimately, we identified a number of RBPs associated with the pathogenesis of HCC, which can be used as potentially prognostic biomarkers and drug targets of HCC patients in the future.

## RESULTS

### Identification of differently expressed RBPs between HCC tumor and normal tissues

**Figure 1:**
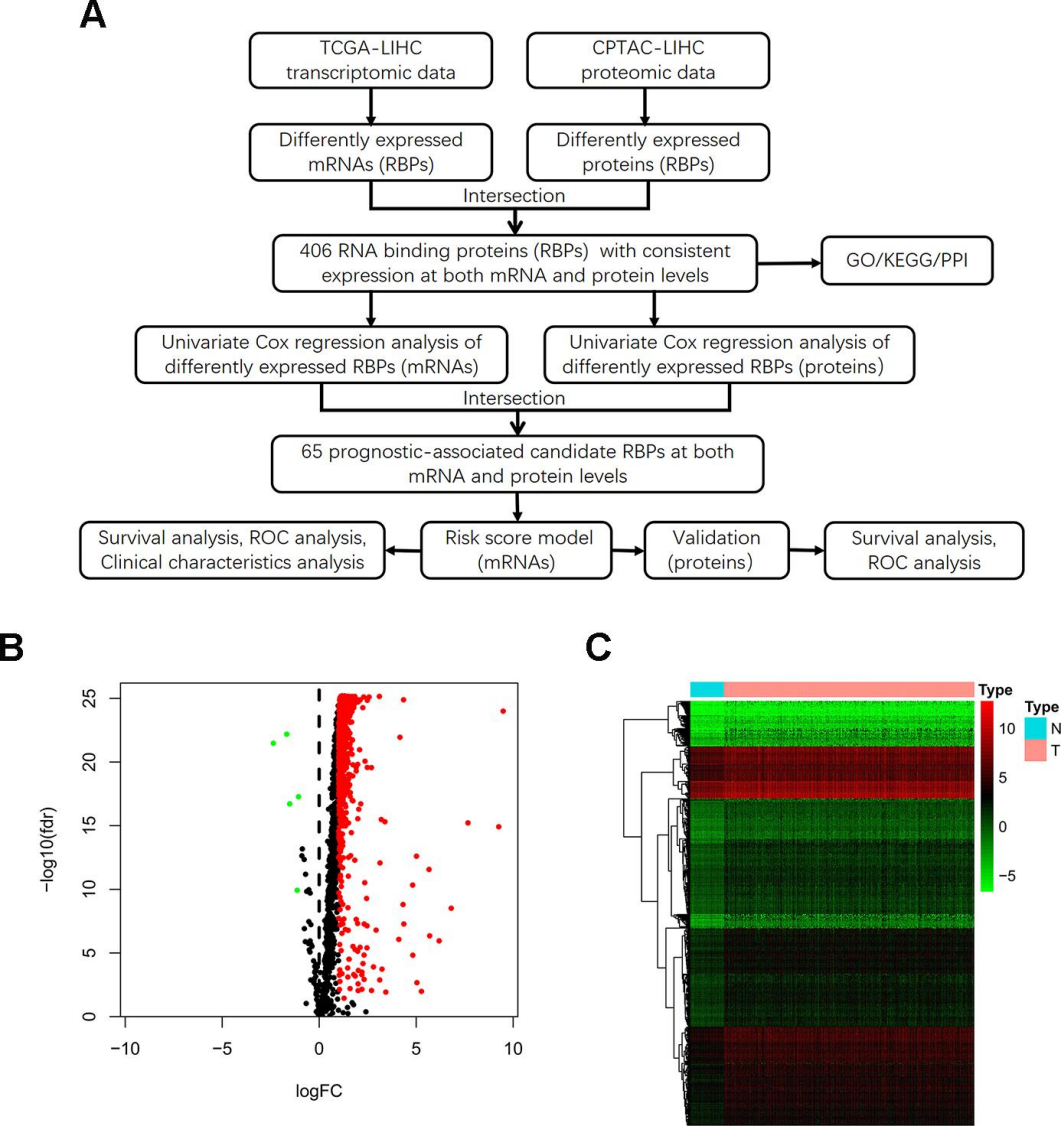
**Identification of differentially expressed RBPs between HCC tumor and normal tissues.** (**A**) Flow chart for analysis of RBPs in HCC. (**B**) Volcano plot for RBPs. Red indicates high expression while green indicates low expression. Black indicates genes that had no differences between HCC tumor and normal tissues. (**C**) Hierarchical clustering analysis of differentially expressed RBPs. The columns indicate samples and the rows are RBPs. Blue represents downregulation while red represents upregulation.

The detailed study design is illustrated in a flow chart (Figure 1A). Initially, we acquired transcriptomic files from the TCGA-LIHC dataset, the analysis of which contained 374 HCC tumor and 50 normal samples. We extracted expression values of 1542 RBPs identified in previous study. Then, we calculated the aberrantly expressed RBPs (|log_2_ FC|≥1, FDR < 0.05). Overall, we identified 557 up-regulated and 5 down-regulated RBPs (Figure 1B). The distribution of the expression levels of these differently expressed RBPs is shown in Figure 1C. Additionally, we downloaded proteomic files, including 159 paired cases, from the CPTAC-LIHC dataset. Thus, we obtained differentially expressed proteins between tumor and normal tissues (|log_2_ FC|≥0, FDR < 0.05). We explored aberrantly expressed RBPs both at the mRNA and protein levels by taking the intersection between transcriptomic and proteomic data. Total 406 RBPs, including 403 up-regulated genes and 3 down-regulated genes, were identified.

### GO and KEGG pathway analyses of differently expressed RBPs

**Figure 2:**
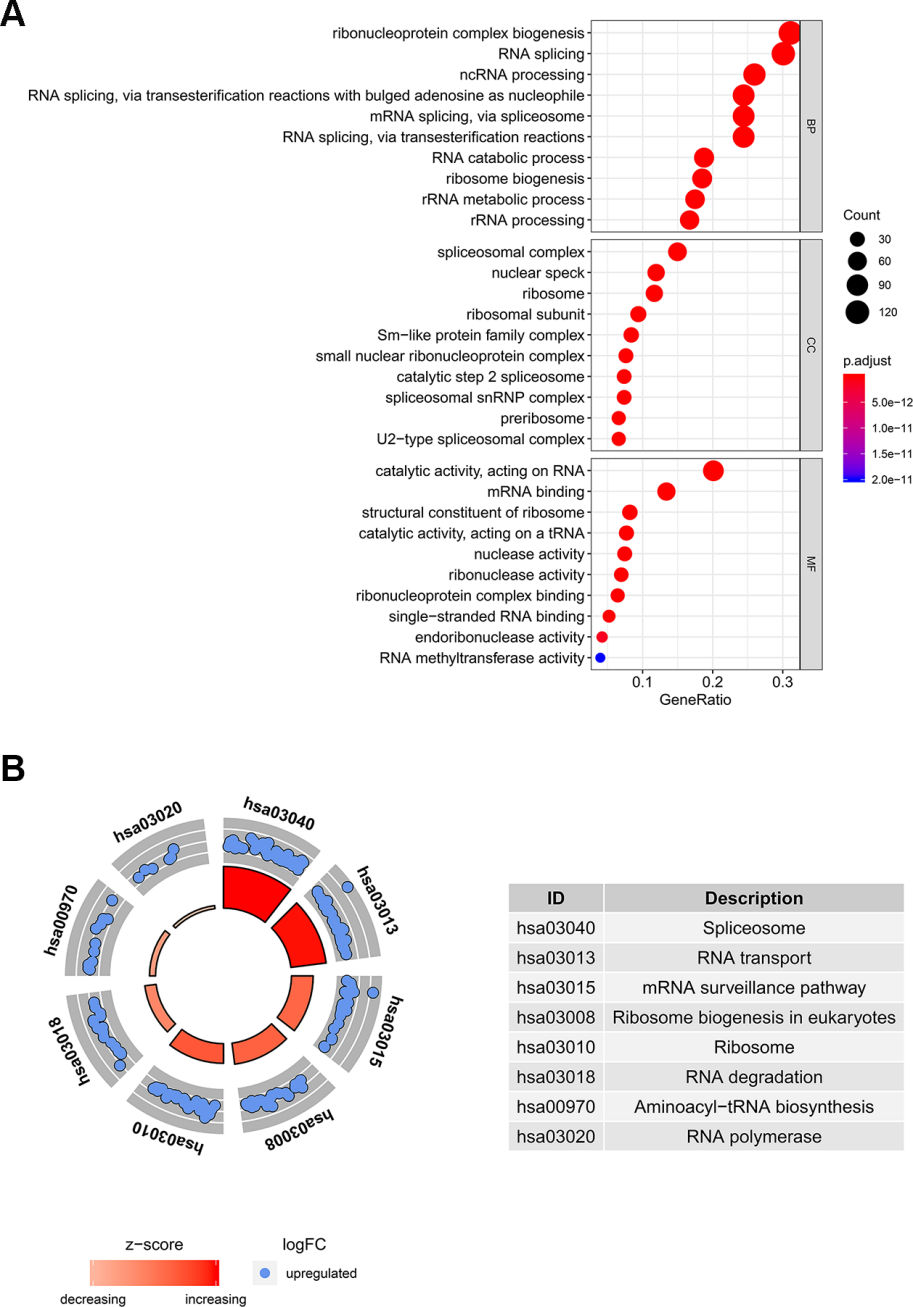
**GO and KEGG pathway analysis of differentially expressed RBPs.** (**A**) The top 10 significantly enriched BPs, CCs and MFs in GO analysis. (**B**) The top 8 significantly enriched pathways in KEGG pathway analysis.

In order to determine the function and mechanisms of these 406 RBPs, we conducted GO enrichment and KEGG pathway analysis. GO enrichment analysis classified RBPs into three functional groups including biological process (BP), cellular component (CC), and molecular function (MF). The top 10 significantly enriched BPs, CCs and MFs are shown in Figure 2A. We found that the 406 differently expressed RBPs were mainly associated with RNA splicing, ribosome biogenesis and RNA binding. Additionally, KEGG pathway analysis indicated that the top 8 significantly enriched pathways included “Spliceosome”, “RNA transport”, “mRNA surveillance pathway”, “Ribosome biogenesis in eukaryotes”, “RNA degradation”, “Aminoacyl−tRNA biosynthesis” and “RNA polymerase” (Figure 2B). These pathways are closely correlated to RNAs maturation, transport, stability and translation. In conclusion, function and pathway enrichment analyses of differently expressed RBPs reflect changes that occur in post-transcriptional gene regulation (PTGR) between tumor and normal tissues.

### Protein-protein interaction (PPI) network construction and key modules analysis

**Figure 3:**
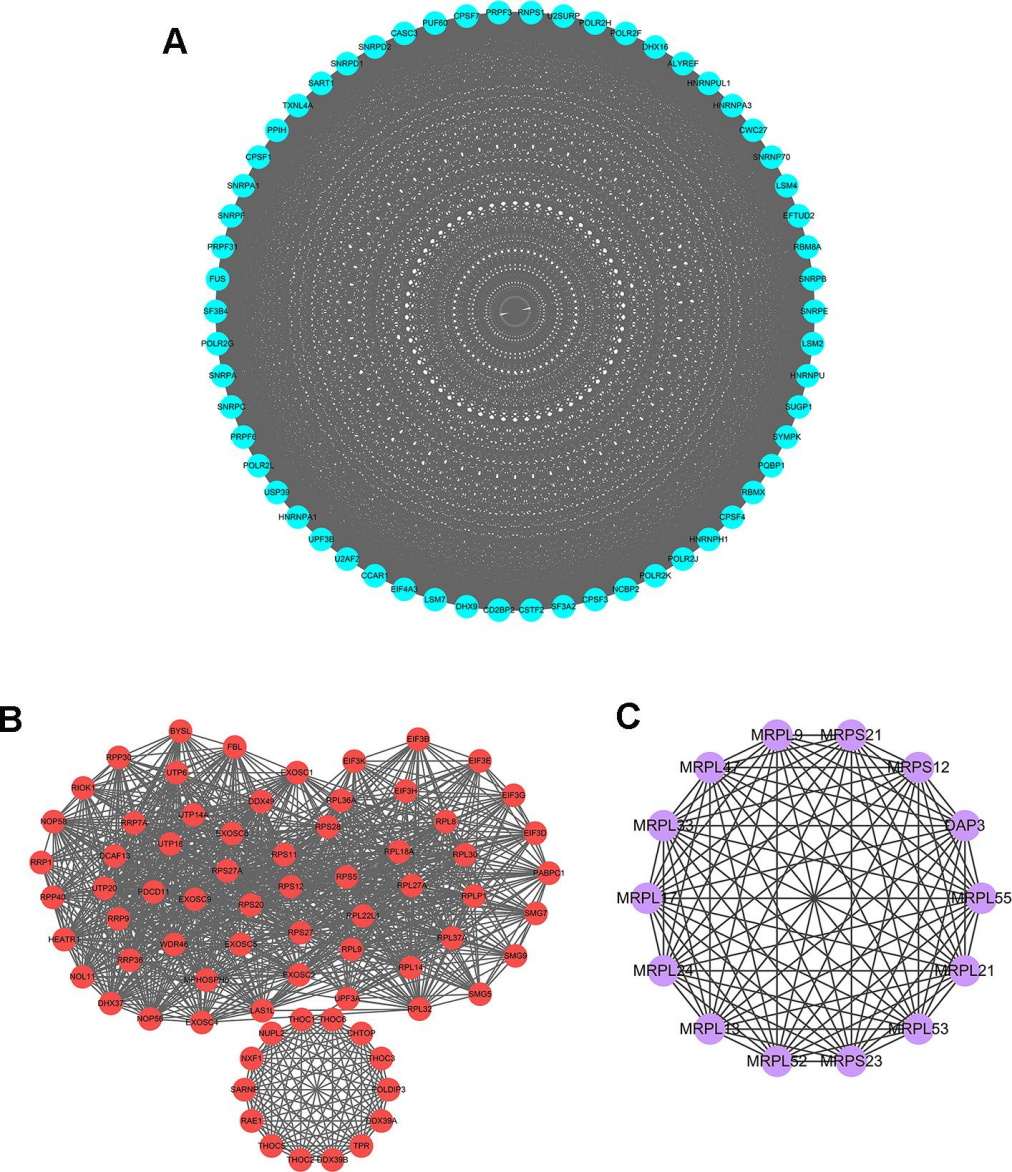
**Key modules inferred from protein-protein interaction (PPI) network of differentially expressed RBPs.** (**A**) Module 1. MCODE score=56.877, Nodes=58, Edges=1621. (**B**) Module 2. MCODE score=30.611, Nodes=73, Edges=1102. (**C**) Module 3. MCODE score=14, Nodes=14, Edges=91.

To further explore the functional protein association networks of differently expressed RBPs in HCC, we submitted the 406 RBPs to the STRING database. We obtained 312 nodes, 4457 edges, and a p-value of PPI concentration <1.0e–16 after setting filter conditions. The top three significant clusters within the PPI network were selected using the Cytoscape software with MCODE plug-in (Figure 3). We also analyzed the function of each module. Pathway enrichment analyses indicated that Module 1 was mainly associated with spliceosome, mRNA surveillance pathway and RNA polymerase (Supplementary Table 1). Module 2 was mainly involved in Ribosome, RNA transport and ribosome biogenesis in eukaryotes, while Module 3 was only related to ribosome pathway (Supplementary Tables 2, 3). The PPI network revealed that RBPs played a vital role in human RNA metabolism, which caused HCC progression.

### Identification of prognosis-related RBPs and construction of risk score model in HCC

**Figure 4:**
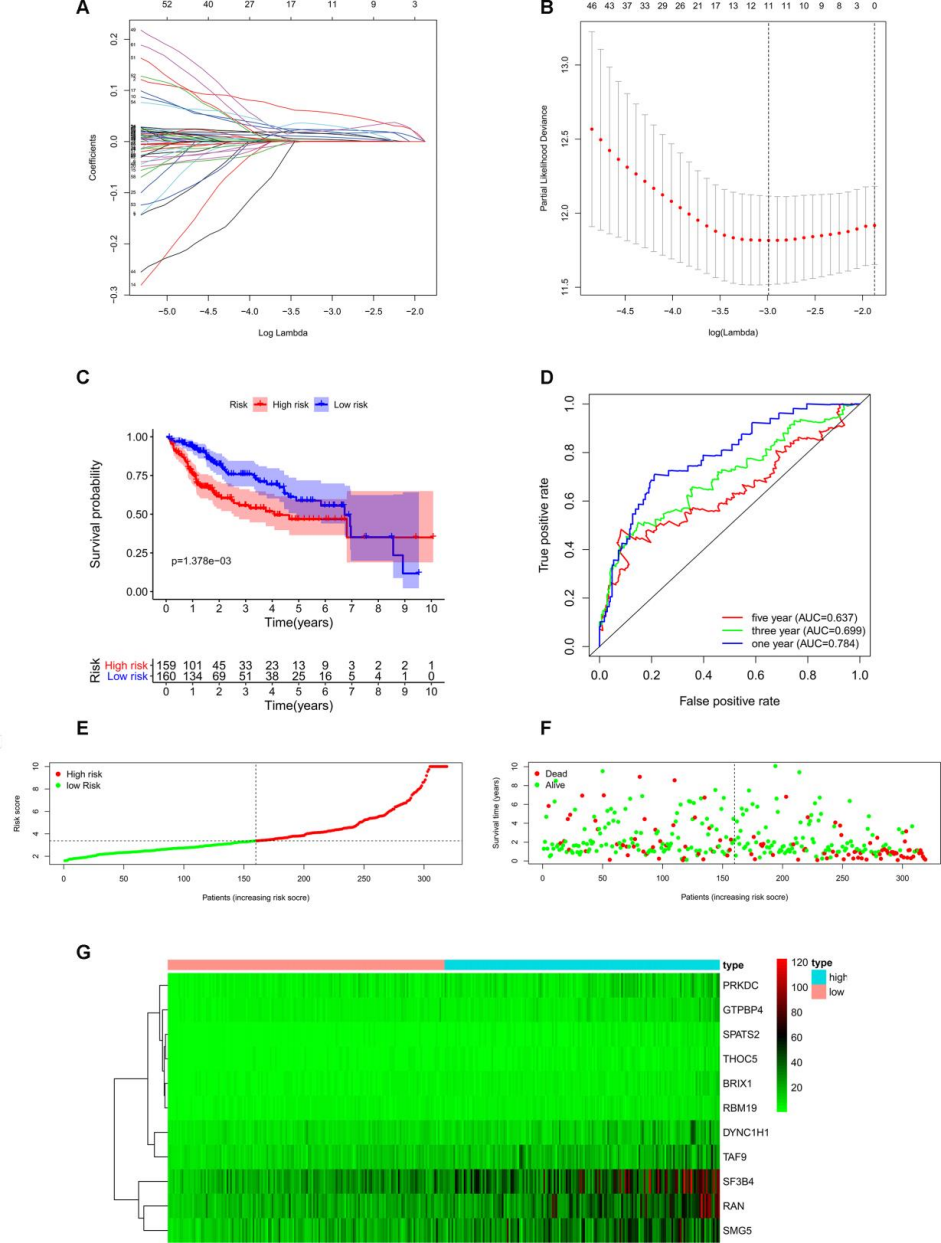
**Construction of risk score model based on prognostic RBPs in the TCGA-LIHC cohort.** (**A**) Each curve represents an RBP, and the best lambda was calculated to minimize mean cross-validated error. (**B**) Cross-validation for tuning parameter selection in the proportional hazards model. (**C**) Kaplan–Meier survival curve of TCGA-LIHC patients. (**D**) Time-dependent ROC curves for predicting OS based on risk scores. (**E**) TCGA-LIHC patients were divided into low-risk group and high-risk group by the median of risk score values. (**F**) Scatter plots show that different risk scores indicate different survival outcomes in the TCGA-LIHC patients. (**G**) The heatmap exhibited the 11 RBPs expression profiles in each TCGA-LIHC patient. Red was defined as high expression and blue indicated as low expression.

In order to investigate the prognostic significance of these differently expressed RBPs, we performed univariate Cox regression analyses both in transcriptomic data and proteomic data. We intersected mRNA-based prognostic RBPs and protein-based prognostic RBPs, and obtained 65 prognosis-associated candidate RBPs. Furthermore, we selected the prognostic RBPs to construct prognostic model by using Lasso Cox regression analysis (Figure 4A, 4B). Overall, 11 RBPs (BRIX1, DYNC1H1, GTPBP4, PRKDC, RAN, RBM19, SF3B4, SMG5, SPATS2, TAF9, and THOC5) were elected to establish a risk score system due to their integrated prognostic relevance. All these RBPs had positive coefficients in the Lasso-penalized Cox regression analysis and severed as independent prognostic factors for overall survival of HCC patients. A risk score formula for each patient was calculated based on expression values of 11 RBPs, and weighed by their estimated regression coefficients in the Lasso Cox regression analysis. We further divided HCC patients into low-risk group and high-risk group based on the median of risk score values. We conducted survival analysis and the results showed that patients in the high-risk group had poorer overall survival compared to patients in the low-risk group (Figure 4C). Furthermore, we carried out the time-dependent receiver operating characteristic (ROC) analysis to assess the prognostic ability. Results indicated that the areas under the ROC curve (AUC) of this risk score model in the TCGA-LIHC cohort were 0.784, 0.699 and 0.637 at 1 year, 3 years and 5 years, respectively (Figure 4D). The risk score model seems to be more precise in the short-term follow-up of HCC patients. We also marked the risk score values of each HCC patient and exhibited their distribution (Figure 4E). Accordingly, there were lower survival time and higher mortality rates in the high-risk group than the low-risk group (Figure 4F). The expression pattern of the 11 prognostic RBPs between the high-risk group and low-risk group is shown in Figure 4G. We found that all 11 prognostic RBPs over expressed in the high-risk group (Figure 4G).

**Figure 5:**
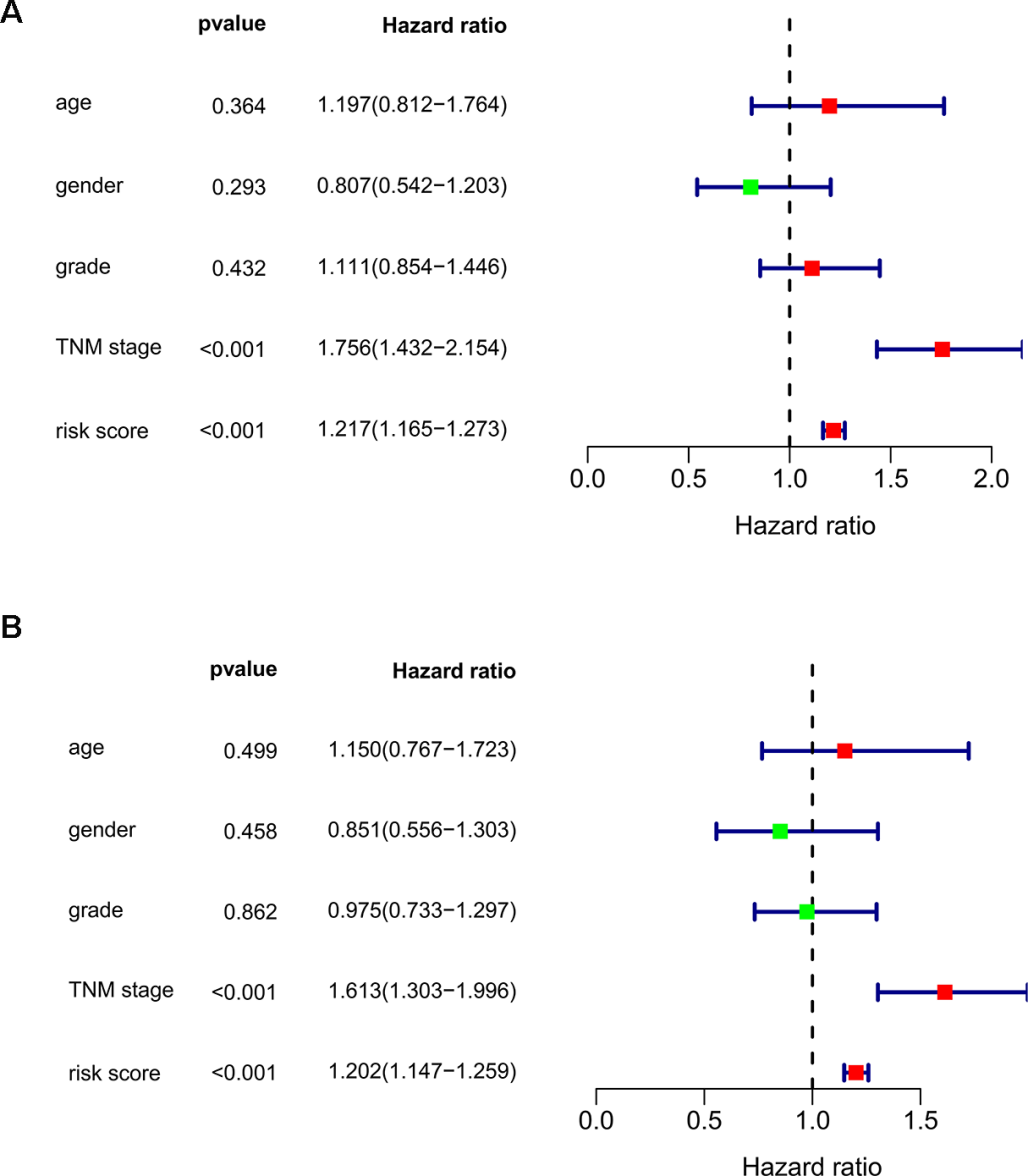
**Assessing the role of prognostic model in survival outcomes.** (**A**) Univariate Cox regression analysis. (**B**) Multivariate Cox regression analysis.

**Figure 6:**
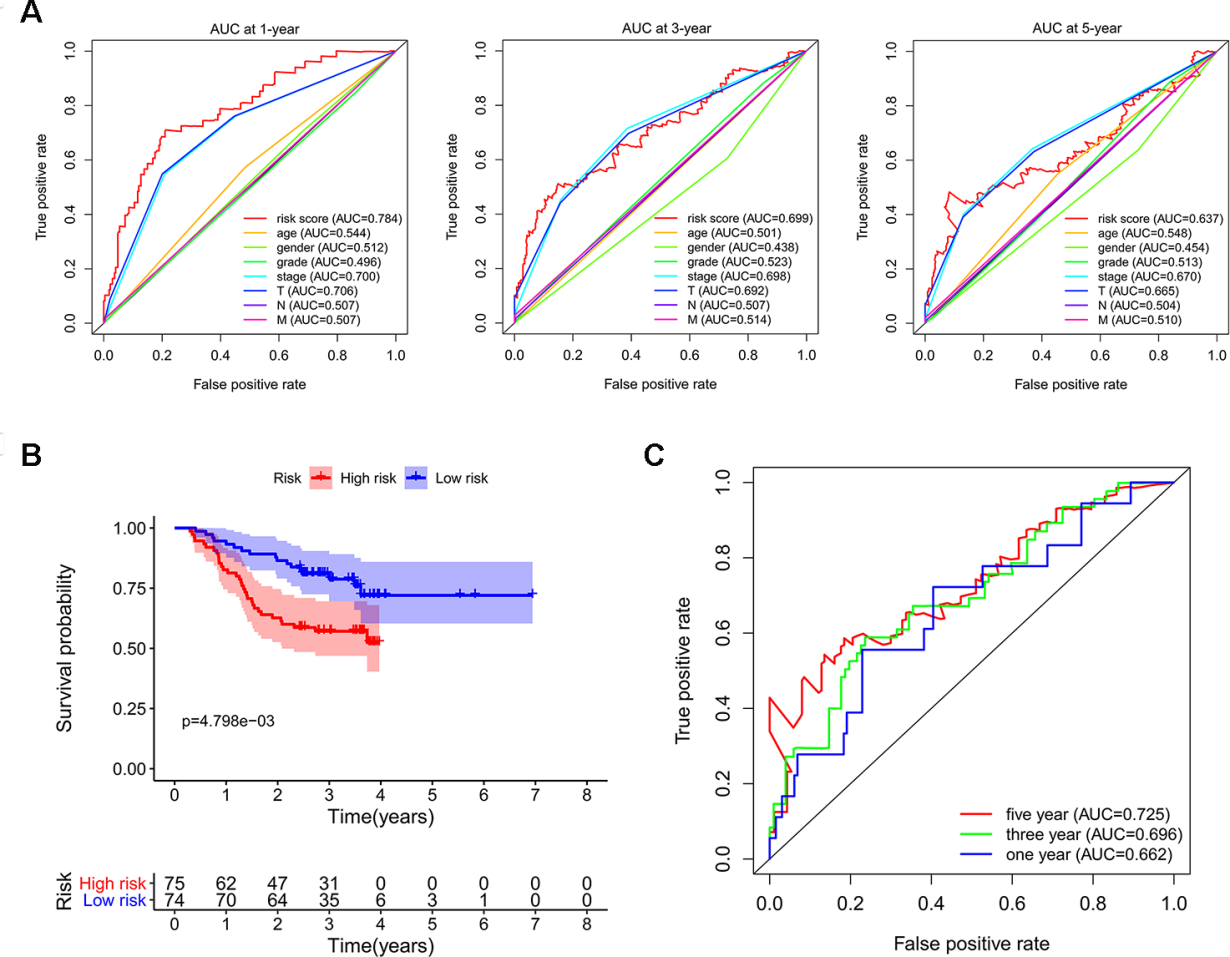
**Validating the reliability of this risk score model.** (**A**) Time-dependent ROC curves for estimating OS based on risk scores and clinical indicators in the TCGA-LIHC cohort. (**B**) Kaplan–Meier survival curve of CPTAC-LIHC patients. (**C**) Time-dependent ROC curves for estimating OS based on risk scores in the CPTAC-LIHC cohort.

We subsequently conducted univariate Cox regression analysis to screen potential indicators correlated with OS in the TCGA-LIHC patients. The results showed that both the TNM stage and risk score were statistically significant (Figure 5A). According to the multivariate Cox regression analysis, the risk score system derived from the expression levels of the 11 RBPs and TNM stage were independent prognostic indicators of OS for HCC patients (Figure 5B). We further assessed the AUC values of available clinical indicators at 1-year, 3-year, and 5-year. These data suggested that the risk score model accurately predicted 1-year and 3-year OS rates of HCC patients compared to other clinical indicators (Figure 6A).

### Validation of the 11 RBPs signature for overall survival prediction in CPTAC-LIHC cohort

Next, we used LIHC patients with reliable prognostic information from the CPTAC database as a validation cohort to confirm the predictive ability of this prognostic model. We calculated the risk scores of each patient based on expression of the previously identified 11 RBPs and regression coefficients for the OS model. Similarly, we subdivided CPTAC-LIHC patients into high-risk group and low-risk group by the median of risk score values. There was a significant difference between the high-risk and the low-risk groups (*p* < 0.05) (Figure 6B). The ROC curve analysis validated that the AUC values for the OS model at 1 years, 3 years and 5 years were 0.662, 0.696 and 0.725, respectively (Figure 6C). These results demonstrated that the risk score model accurately predicted prognosis of CPTAC-LIHC patients in long-term follow-up.

### Relationships between the prognostic RBPs and clinicopathological features

**Figure 7:**
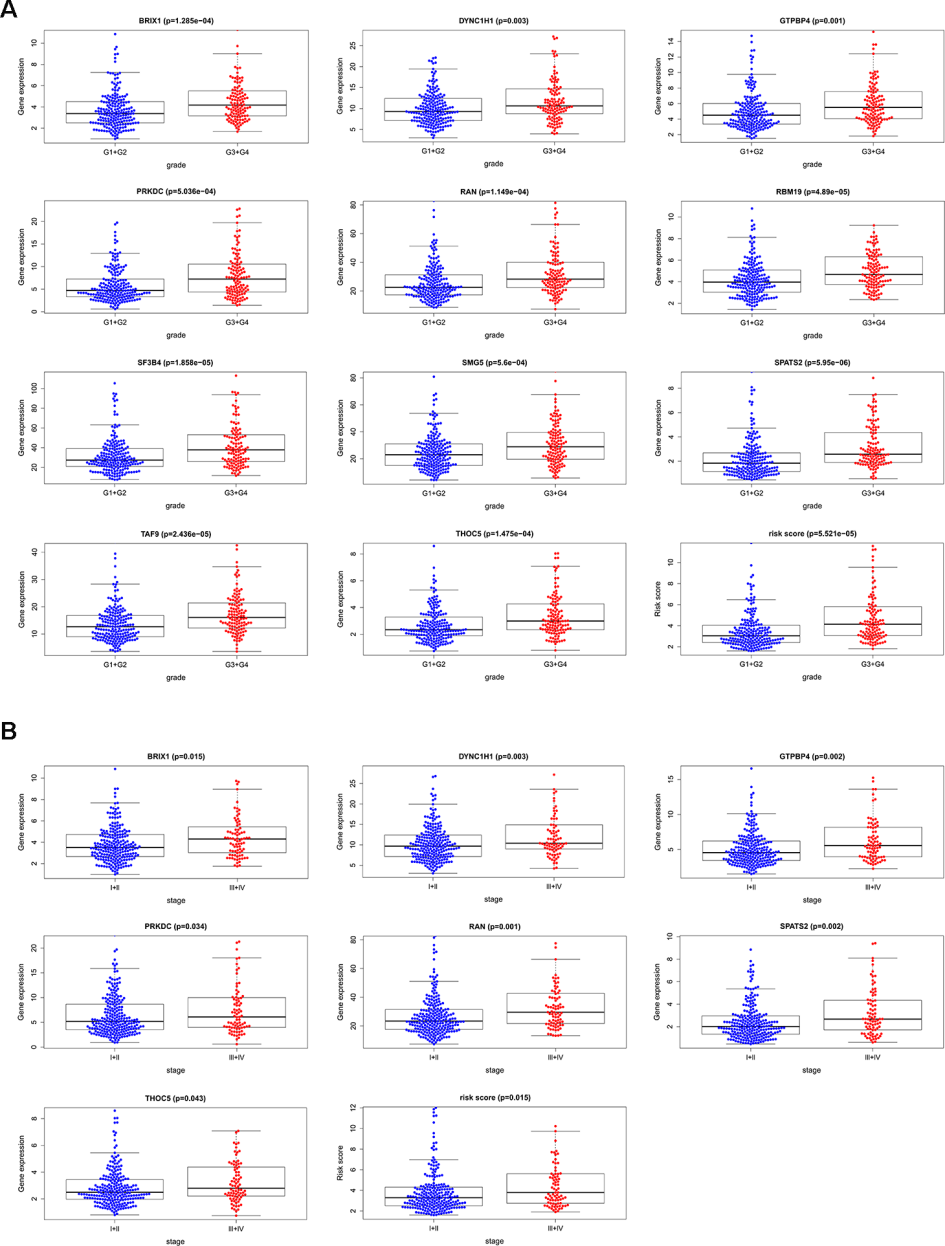
**The relevance between 11 prognostic RBPs and clinicopathologic features.** (**A**) The relationship between 11 prognostic RBPs and tumor grade. (**B**) The relationship between 11 prognostic RBPs and TNM stage.

We analyzed the relevance between the 11 prognostic RBPs and clinicopathological features, such as tumor grade and TNM stage, in order to explore the differentially diagnostic capability of the prognostic signature. All 11 RBPs (BRIX1, DYNC1H1, GTPBP4, PRKDC, RAN, RBM19, SF3B4, SMG5, SPATS2, TAF9, and THOC5) were found to be significantly higher in patients with advanced tumor grade (*p* < 0.05). Only seven RBPs (BRIX1, DYNC1H1, GTPBP4, PRKDC, RAN, SPATS2, THOC5) expressed at higher levels in patients with advanced TNM stage (*p* < 0.05) (Figure 7). However, the risk score model was related to both tumor grade and TNM stage.

### Genomic information and drug-RBPs interaction of the eleven RBPs

**Figure 8:**
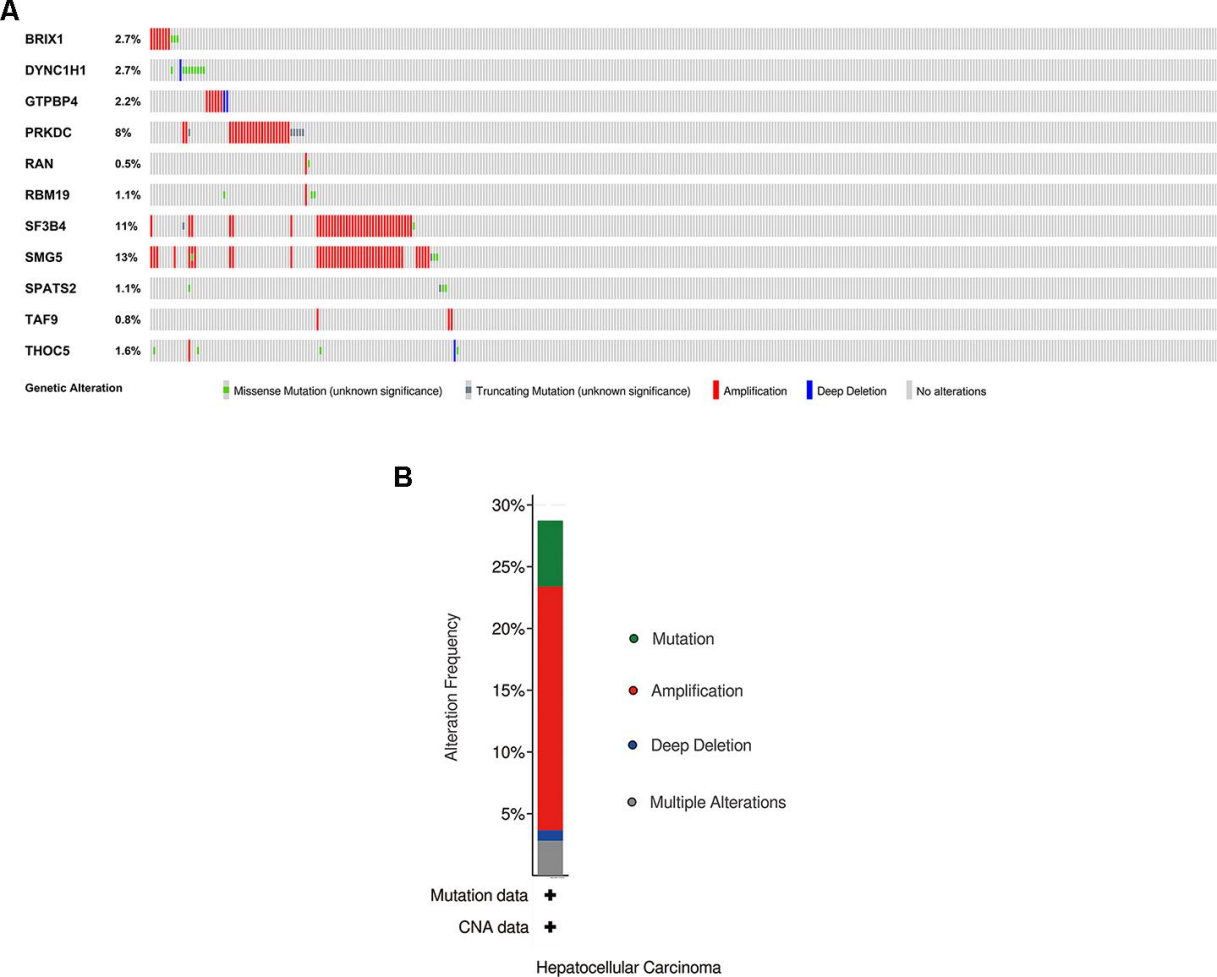
**Exploration of 11 prognostic RBPs as potentially therapeutic targets for HCC patients.** (**A**) Genetic alterations of each prognostic RBP. (**B**) An overview of all genomic changes for 11 prognostic RBPs.

We explored the genetic alterations of these prognostic RBPs in the cBioPortal database. Figure 8A, 8B presents data on genetic alterations including missense mutation, truncating mutation, amplification, deep deletion and no alterations. In total, 11 RBPs were altered in about 29% (106/366) of queried samples in HCC (TCGA, Firehose Legacy). Among these, SMG5, SF3B4 and PRKDC are the top 3 most significantly altered genes in HCC samples.

The 11 prognostic RBPs served as promising targets for HCC patients. We explored the drug-gene interactions through the DGIdb database. Among these 11 RBPs, only PRKDC and RAN were identified as chemotherapeutic targets. Table 1 shows that most of the drugs were inhibitors of PRKDC. Therefore, we hypothesize that the additional 9 genes (BRIX1, DYNC1H1, GTPBP4, RBM19, SF3B4, SMG5, SPATS2, TAF9, and THOC5) might be novel targets in the future.

**Table 1:** Candidate drugs targeting prognostic RBPs.

**Number**	**Gene**	**Drug**	**Interaction type**	**Sources**	**Score^a^**
1	PRKDC	CHEMBL1086377	inhibitor	GuideToPharmacologyInteractions	1
2	PRKDC	CHEMBL188678	inhibitor	GuideToPharmacologyInteractions	1
3	PRKDC	PI-103	inhibitor	GuideToPharmacologyInteractions	1
4	PRKDC	CHEMBL1081312	inhibitor	GuideToPharmacologyInteractions	1
5	PRKDC	WORTMANNIN	inhibitor	GuideToPharmacologyInteractions	1
6	PRKDC	CC-115	inhibitor	ChemblInteractions	1
7	PRKDC	SF-1126	—	TdgClinicalTrial, DrugBank	2
8	RAN	CHEMBL384759	—	DrugBank	2

^a^ The score is the combined number of database sources and PubMed references supporting a given interaction.

### External validation of prognostic RBPs expression in the HPA database

**Figure 9:**
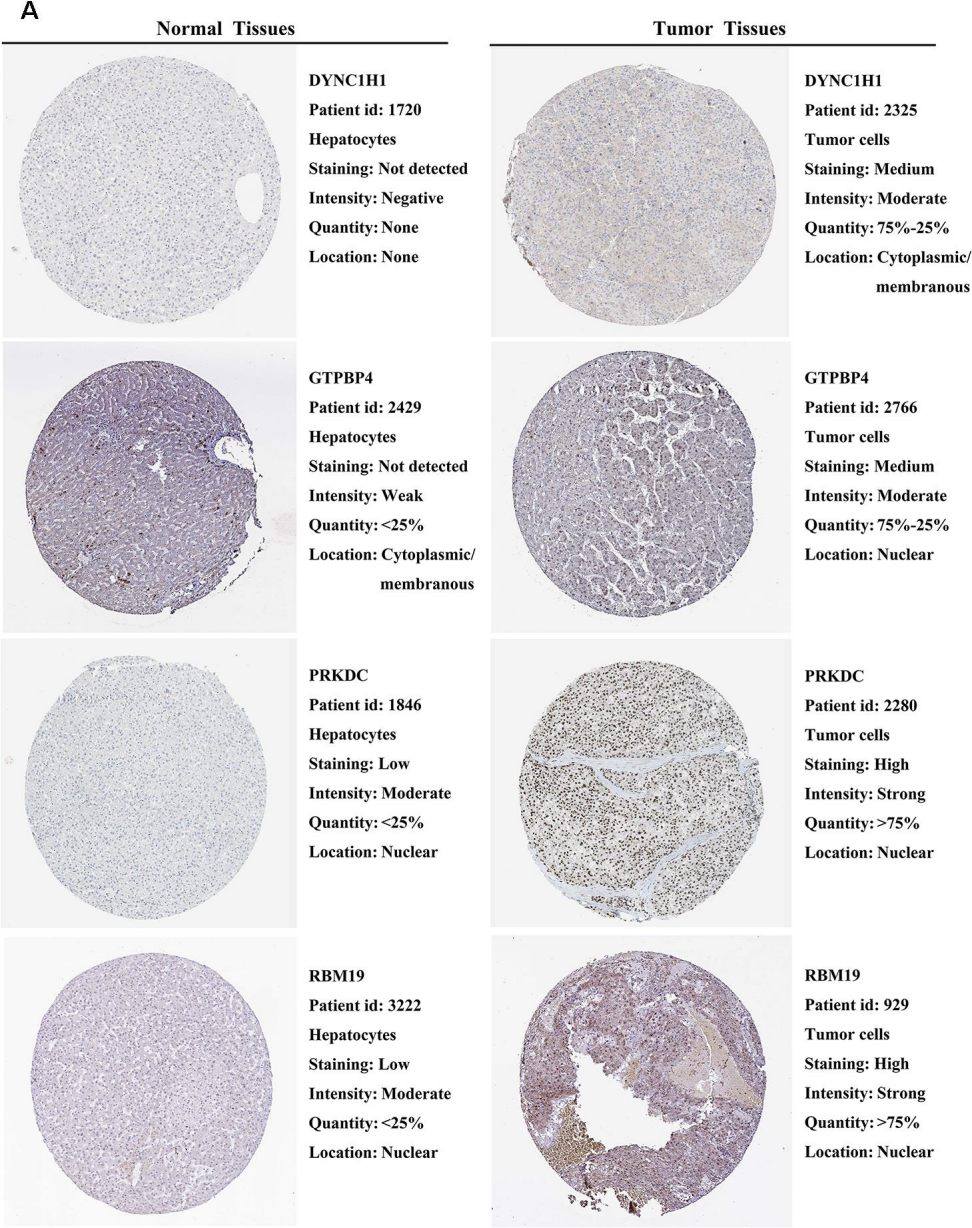
**Validation of prognostic RBP expression in the HPA database.** (**A**) Representative immunohistochemical staining of HCC primary tumor tissues and normal liver tissues.

**Figure 10:**
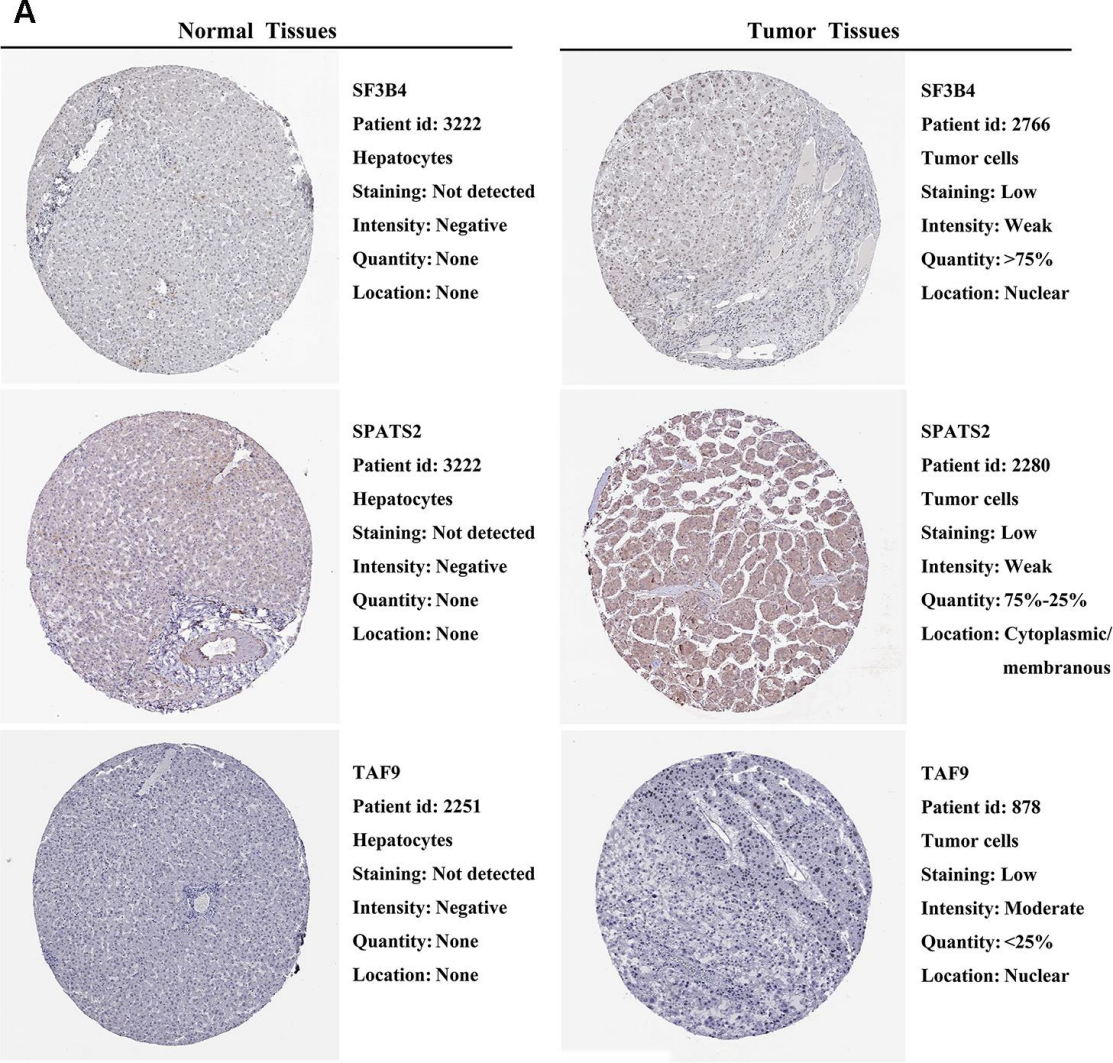
**Validation of prognostic RBP expression in the HPA database.** (**A**) Representative immunohistochemical staining of HCC primary tumor tissues and normal liver tissues.

We further explored the expression of the 11 prognostic RBPs in the Human Protein Atlas (HPA) database. The immunohistochemistry (IHC) results demonstrated that DYNC1H1, GTPBP4, PRKDC, RBM19, SF3B4, SPATS2 and TAF9 were significantly increased in HCC tumor cells compared to normal hepatocytes (Figures 9, 10). However, IHC staining of BRIX1, RAN, SMG5 and THOC5 was missing and was pending further analysis.

### Concrete clinical analysis and functional study of SPATS2 in HCC

**Figure 11:**
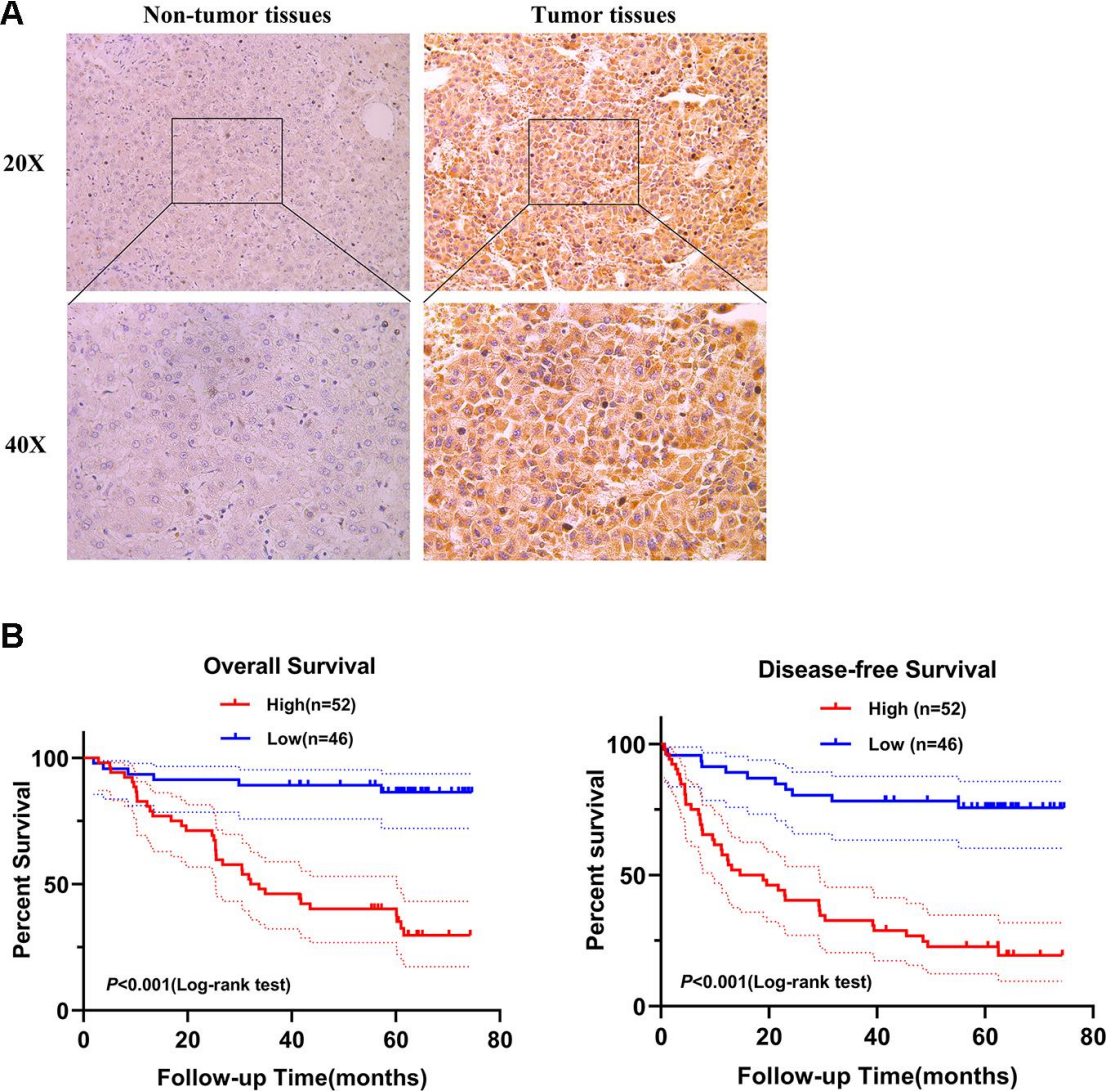
**SPATS2 is upregulated in HCC primary tumor tissues and correlates with poorer prognosis.** (**A**) Representatives of SPATS2 staining in a pair of tumor tissue and adjacent non-tumor tissue. The boxed regions are amplified as images below. (**B**) Kaplan-Meier plots of overall survival and disease-free survival.

Next, we selected SPATS2 as a representative prognostic RBP for validation. We determined the expression of SPATS2 by immunohistochemistry across 98 pairs of HCC samples acquired from Peking University Cancer Hospital and Institute. SPATS2 was notably up-regulated in HCC tumor tissues compared to non-tumor tissues (Figure 11A). Our results indicated that HCC patients with high expression of SPATS2 had lower overall survival rates and disease-free survival rates compared to the low expression group (p < 0.001, Figure 11B). Next, we analyzed the correlation between up-regulated expression of SPATS2 and clinicopathological features of HCC patients. Our results showed that SPATS2 up-regulation was significantly correlated to tumor size (p = 0.015) (Table 2). Univariate Cox analysis also indicated that there was significant correlation between tumor size, serum AFP levels, microscopic vascular invasion, Edmondson-Steiner grade, SPATS2 up-regulation and overall survival (*p* < 0.05). On the other hand, the tumor nodule number and distant metastasis were not significantly associated with overall survival (*p* > 0.05). In order to rule out the possibility that the above single factor variable was a covariate, further multivariate Cox analysis manifested that the tumor size, serum AFP levels, Edmondson-Steiner grade and SPATS2 up-regulation were independent prognostic factors for overall survival in HCC (*p* < 0.05, Table 3).

**Table 2:** Association analysis between SPATS2 up-regulation in tumor and clinicopathologic characteristics of HCC patients.

**Clinicopathologic Characteristics**	**Total**	**SPATS2 up-regulation**	***P* value**
**Tumor size (cm)**			0.015
<=5	60	26 (43.3%)	
>5	38	26 (68.4%)	
**Serum AFP level (ng/ml)**			0.286
<=200	65	32(49.2%)	
>200	33	20 (60.6%)	
**Tumor nodule number**			0.230
Solitary	78	39 (50.0%)	
Multiple	20	13 (65.0%)	
**Microscopic vascular invasion**			0.383
Absent	66	33 (50.0%)	
Present	32	19 (59.4%)	
**Edmondson-Steiner grade**			0.744
I+II	76	41 (53.9%)	
III+IV	22	11 (50.0%)	
**Distant metastasis**			0.436
Absent	93	48(51.6%)	
Present	5	4(80.0%)	

**Table 3:** Univariate and multivariate Cox analyses of prognostic variables in HCC patients.

**Clinicopathologic characteristics**	Univariable analysis*		Multivariable analysis*	
	**HR (95%CI)**	***P* value**	**HR (95%CI)**	***P* value**
Tumor size	2.539(1.366~4.717)	0.003	2.521(1.305~4.872)	0.006
Serum AFP level	2.631(1.420~4.876)	0.002	2.366(1.270~4.407)	0.007
Tumor nodule number	1.864(0.948~3.663)	0.071		
Microscopic vascular invasion	2.389(1.291~4.420)	0.006		
Edmondson-Steiner grade	2.068(1.070~3.994)	0.031	2.654(1.318~5.341)	0.006
Distant metastasis	2.637(0.938~7.410)	0.066		
SPATS2 up-regulation	1.285(1.121~1.474)	<0.001	1.302(1.115~1.521)	0.001

*Cox regression model was used in the analyses;

HR, Hazards ratio; CI, confidence interval.

**Figure 12:**
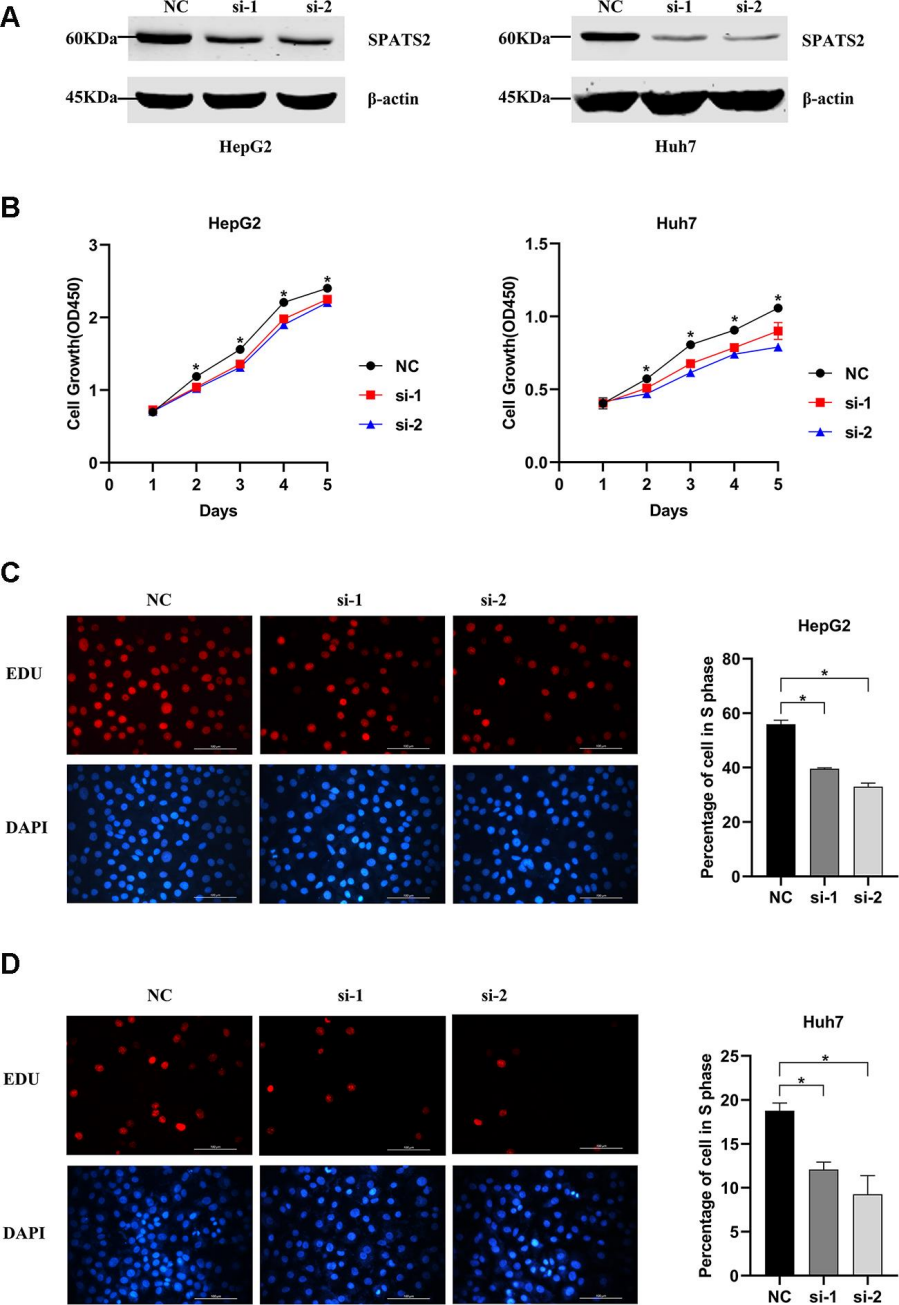
**Knockdown of SPATS2 inhibits cell proliferation of HCC cells.** (**A**) SPATS2 expression in transfected HepG2 and Huh7 cells is confirmed by western blotting. (**B**) CCK8 assay is used to compare cell growth between SPATS2 knockdown cells and negative control cells. (*p < 0.05). (**C**, **D**) EdU assay is used to compare cell proliferation between SPATS2 knockdown cells and negative control cells. (*p < 0.05).

**Figure 13:**
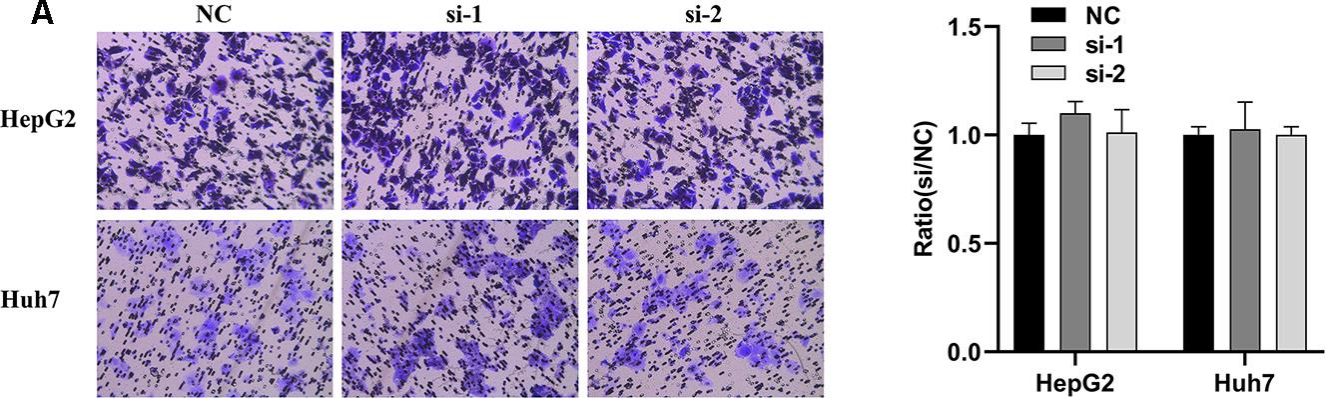
**Knockdown of SPATS2 does not inhibit cell migration of HCC cells.** (**A**) Representative and summary of cell migration assays performed with SPATS2 knockdown cells and negative control cells.

In order to explore whether SPATS2 has an effect on the function of HCC cells, we transiently transfected siRNAs into HepG2 cells and Huh7 cells (Figure 12A). According to results of the CCK8 assay, knockdown of SPATS2 inhibited cell growth compared to the negative control (Figure 12B, *p* < 0.05). Consistently, EdU cell proliferation assay indicated that SPATS2 depletion led to a significant reduction of S phase cells (Figure 12C, 12D, *p* < 0.05). However, there were no significant changes in the migration assay between the negative control and siRNAs (Figure 13A, *p* > 0.05). In conclusion, our results proved that SPATS2 depletion inhibited proliferation of HCC cells.

## DISCUSSION

In this study, we identified RBP-based molecular biomarkers that can predict overall survival of HCC patients. Metastasis and recurrence after resection are major limitations to the treatment of HCC patients [3]. Therefore, prognostic assessment is a crucial step. In clinic, prognostic evaluation incorporates not only tumor stage and cancer-related symptoms, but also the degree of liver function impairment [18]. Transcriptome sequencing and mass-spectrometry technologies help us decode transcriptomic and proteomic changes in the development of cancer, which are beneficial for identifying promising biomarkers for cancer diagnosis, treatment, and prognosis [19–22].

RBPs bind all types of RNAs through RNA-binding domains (RBDs) that form steady secondary and tertiary structures [13]. The common RBDs include RNA recognition motif (RRM), K-homology domain (KH), Zinc finger domain (ZNF), double stranded RNA binding domain (dsRBD), cold shock domain (CSD), La motif, Piwi/Argonaute/Zwille (PAZ) domain, and Piwi domain [7]. RBPs with multiple modules define the fundamental structural unit that is responsible for biological function [7]. RBPs are known for their role in many regulatory processes, including post-transcriptional regulation of RNA stability, splicing, editing, maturation, translation and localization, which ultimately impacts gene expression [9]. Dysregulation of RBPs contributes to transcriptomic imbalance in tumor cells and drives tumorigenicity, including in HCC. NELFE, an oncogenic RBP, is able to activate MYC signaling by binding directly to MYC or its targets and enhances HCC development [23]. It has been reported that HuR, a member of the ELAV family of RNA-binding proteins, is associated with MAT2A 3’ UTR and enhances MAT2A mRNA stability, whereas AUF1 is associated with MAT1A 3’UTR and decreases MAT1A mRNA stability [24]. HuR is an important regulator of liver de-differentiation, development, proliferation and carcinogenesis through the post-translational regulation of MAT1A and MAT2A mRNAs [24]. Another study indicates that the liver-specific lncRNA HULC is discovered to be an IGF2BP substrate and destabilizes through CNOT1-mediated deadenylation recruited by IGF2BP1 [25]. Moreover, RPS3 as an RBP is frequently up-regulated in human HCC [26]. A study unveils a novel role of RPS3 in facilitating hepatocarcinogenesis through stabilizing SIRT1 mRNA [26].

Our study integrated TCGA-LIHC RNA sequencing data and CPTAC-LIHC proteomic data in order to select differentially expressed RBPs between HCC tumor tissues and normal tissues. Total 406 RBPs were observed to have consistent changes at both the transcriptional and protein levels. Then, we conducted GO enrichment and KEGG pathway analysis, and constructed a protein-protein interaction network. The key modules displayed that the differentially expressed RBPs were greatly associated with spliceosome, mRNA surveillance pathway, RNA transport and ribosome biogenesis. In addition, we discussed the potential clinical application of these RBPs. Univariate Cox regression analysis identified 65 prognostic-associated candidate RBPs. With Lasso Cox regression analysis, we selected 11 RBPs (BRIX1, DYNC1H1, GTPBP4, PRKDC, RAN, RBM19, SF3B4, SMG5, SPATS2, TAF9, and THOC5) to build a risk score model. The TCGA-LIHC patients were further assigned to low-risk and high-risk groups based on the risk score median and patients in high-risk group had poorer overall survival compared to patients in the low-risk group. The ROC curve analysis revealed that these 11 RBPs signature had a better prognostic capability for HCC patients. Simultaneously, we validated the conclusion in CPTAC-LIHC patients. This implied that we initiatively performed the first transcriptomic and proteomic characterization of RBPs-based HCC. The combined analyses of RNA sequencing and mass spectrometry generated more comprehensive knowledge that united cancer ‘‘genotype’’ with ‘‘phenotype’’ through functional proteomics and signaling networks [27]. In addition, both types of data wielded a more integrated view of tumor biology that helped patients identify more effective treatments than they would by using genomics alone [28].

On the other hand, each RBP in the prognostic signature was evaluated for its relationship with clinicopathological features. All 11 RBPs (BRIX1, DYNC1H1, GTPBP4, PRKDC, RAN, RBM19, SF3B4, SMG5, SPATS2, TAF9, and THOC5) were expressed at significantly higher levels in patients with advanced tumor grade. Only seven RBPs (BRIX1, DYNC1H1, GTPBP4, PRKDC, RAN, SPATS2, THOC5) were expressed higher in patients at advanced TNM stage. We conducted in-depth exploration of altered genetic information for the 11 prognostic RBPs, including missense mutation, truncating mutation, amplification and deep deletion. Taken together, these were abnormally expressed in every gene. PRKDC, SF3B4 and SMG5 ranked top three with high amplification ratio. Among these RBPs, the known drug targets were PRKDC and RAN. It has been reported that oncogenic RBPs such as GTPBP4, PRKDC, RAN, SF3B4, SMG5, SPATS2, TAF9, and THOC5 promoted HCC cell proliferation, apoptosis, migration and invasion, which led to tumorigenesis and poor overall survival. These findings may contribute to the development of novel biomarkers for treatment and prognosis of HCC patients.

GTPBP4, also known as NOG1 or NGB, is a novel member of the GTPase family and resembles the α subunits of the heterotrimeric G-proteins [29]. The nucleolar localization of NOG1 is associated with a precursor particle to the 60S subunit as well as co-precipitation with the 60S precursor RNAs [30]. It has been reported that GTPBP4 is a p53 interactor and knockdown of GTPBP4 induces p53 accumulation and activation in absence of nucleolar disruption [31]. In addition, high expression of GTPBP4 is significantly correlated with reduced survival in breast cancer, colorectal carcinoma and HCC [31–33]. Knockdown of GTPBP4 delays cell proliferation, induces cell cycle arrest in G2/M period and promotes apoptosis in HCC cell lines [33].

PRKDC/DNA-PKcs encodes the catalytic subunit of the DNA-dependent protein kinase (DNA-PK). Ku heterodimer protein and DNA-PKcs collectively comprise the DNA-PK [34]. Once double-strand DNA breaks, Ku binds to DNA ends, recruits and activates DNA-PKcs which has a diverse array of nuclease activities, and initiates the classical non-homologous end-joining (cNHEJ) [34, 35]. Ku also drives the assembly of DNA-PKcs on a wide range of cellular RNAs, including the U3 small nucleolar RNA, which is essential 18S rRNA processing [36, 37]. Additionally, DNA-PK has an RNA-dependent, cNHEJ-independent function during ribosome biogenesis that requires kinase activity of DNA-PKcs [37]. Based on these features, DNA-PKcs often protects tumor cells from DNA damage that derives from chemotherapy and radiotherapy [38–41]. Specifically, DNA-PKcs promotes proliferation, inhibits apoptosis and facilitates DNA-repair of HCC cells independent of p53 [42]. Clinically, DNA-PKcs is a candidate driver gene of hepatocarcinogenesis and elevated DNA-PKcs identifies HCC patients with treatment-resistance, whereas elevation of activated pDNA-PK independently predicts poor survival [43]. Furthermore, DNA-PKcs is summarized as a promising therapeutic target in human HCC [44].

RAN (Ras-related nuclear protein), a member of the RAS superfamily of small GTPases, is essential for translocation of RNA and proteins through the nuclear pore complex during cell cycle [45, 46]. For example, miRNAs are initially produced from pre-miRNAs. Exportin-5 in a complex with RAN recognizes and binds to the pre-miRNA molecule, exporting it to the cytoplasm [47]. In HCC, genetic variations in miRNA processing genes (rs1057035 in DICER1, rs3803012 in RAN, and rs10773771 in PIWIL1) have an effect on miRNA biogenesis [48]. Finally, RAN rs3803012 AG/GG variant genotypes increased the risk of HBV persistent infection [48]. It has also been reported that a panel of 5 genes (*TAF9, RAMP3, HN1, KRT19,* and *RAN*) showed the strongest prognostic relevance. Among these genes, RAN was up-regulated in poor prognosis of HCC [49].

SF3B4 is a member of the splicing factor 3b (SF3b) complex family that is essential for accurate excision of introns from pre-messenger RNAs [50]. A study demonstrated that SF3B4 overexpression triggered SF3b complex to splice the tumor suppressor KLF4 transcript to nonfunctional skipped exon transcripts. This contributed to malignant transformation and growth of hepatocytes through transcriptional inactivation of p27/Kip1 and simultaneously activation of Slug genes [51].

SMG5 is involved in nonsense-mediated mRNA decay (NMD) [52]. NMD degrades transcripts with premature or aberrant translation termination codons and thereby prevents the synthesis of C-terminally truncated proteins [53]. The central NMD factor UPF1 recruits the endonuclease SMG6 and the deadenylation-promoting SMG5/7 complex to participate in endo- and exonucleolytic decay [54]. Up-regulation of SMG5 is also associated with poor prognosis in HCC [55].

TAF9 encodes a smaller subunit of TFIID, mediates transcriptional activation of p53-mediated transcriptional activity and leads to p53-dependent growth arrest in fibroblasts [56]. Once other components of the p53 pathway is mutated, this coactivator of p53-mediated transcription results in disease [57]. On the other hand, the complex STAGA (SPT3-TAF9-GCN5-acetylase) recruits a mediator to the MYC oncoprotein in order to stimulate transcription and cell proliferation [58]. TAF9 is also up-regulated in HCC patients with poor prognosis [49].

THOC5 is a member of the THO complex and is involved in processing and transport of mRNAs [59]. THOC5 influences more than 90% of growth factor/cytokine-induced genes [60]. For instance, THOC5 is indispensable for processing of mRNAs (Sox9 and Ascl2) that are induced by Wnt signaling [61]. THOC5 contributes to pathway from leukemogenic oncogenes and stem cell chemokines to RNA processing [59]. Moreover, 50% depletion of THOC5 in the HCC cell lines Huh7 and HepG2 induces apoptosis, and THOC5 expression is enhanced in 78% of cytological differentiation grading G2 and G3 tumor in primary HCC [62].

SPATS2 is a novel candidate biomarker for squamous cell carcinoma [63]. Interestingly, high expression of SPATS2 is associated with poor prognosis in liver cancer as well [64]. In our study, we demonstrate that up-regulation of SPATS2 is an independent prognostic factor for overall survival in HCC. Furthermore, we find that SPATS2 upregulation is significantly correlated with tumor size. Functional analysis indicates that SPATS2 depletion inhibits cell proliferation rather than migration in HCC cell lines. Therefore, we conclude that SPATS2 accelerates cell proliferation in hepatocarcinogenesis.

Although our study indicates that RNA-binding proteins prominently contribute to the progress of HCC, there are several limitations. It is still not fully understood that the detailed biological functions and molecular mechanism of the 11 RBPs contribute to hepatocellular carcinogenesis. Hence, there should be further exploration of potential mechanisms in the future. Meanwhile, most of drugs targeting RBPs are PRKDC inhibitors that have not been used in clinical medicine. More studies and clinical trials are needed to identify effective drugs for targeting prognostic RBPs in HCC patients. Valuable insights will be pushed into the individualized therapy based on high-throughput technology to suit the circumstance of each patient with HCC, as well.

## MATERIALS AND METHODS

### Data acquisition and analysis

A panel of 1542 RNA-binding proteins (RBPs) were included in this study [9]. The RNA sequencing data and corresponding clinical data were downloaded from the Cancer Genome Atlas database (TCGA, https://portal.gdc.cancer.gov/) [21]. Then we extracted RBP expression from TCGA-LIHC dataset including 374 hepatocellular carcinoma (HCC) samples and 50 normal samples for subsequent analysis. All raw data were preprocessed by the Limma package in R software (Version 3.5.1). We recognized the differentially expressed RBPs between normal and tumor tissues with the following criteria: |log_2_ FC|≥1 and FDR < 0.05. The proteomic data and corresponding clinical data, including 159 paired HCC samples, were acquired from the CPTAC Data Portal (https://cptac-data-portal.georgetown.edu/cptacPublic/). Data used in this publication was generated by the National Cancer Institute Clinical Proteomic Tumor Analysis Consortium (CPTAC) [22]. The differentially expressed proteins between tumor and normal tissues were identified using the following criteria: |log_2_ FC|≥0 and FDR < 0.05. Unsupervised clustering analysis was performed by using the “pheatmap” package in R software.

### KEGG pathway and GO enrichment analyses

GO enrichment analysis and Kyoto Encyclopedia of Genes and Genomes (KEGG) pathway analysis were performed in R software. The GO analysis terms included cellular component (CC), molecular function (MF) and biological process (BP). *P* < 0.05 was used as the threshold for statistical significance.

### Protein-protein interaction (PPI) network construction and key modules analysis

We submitted the differentially expressed RBPs to the STRING database (http://www.string-db.org/) to build PPI network [65]. The TSV file generated by STRING database was imported into Cytoscape 3.7.1 software in order to identify the key modules by using Molecular Complex Detection (MCODE) plug-in with both MCODE score and node counts more than 10. MCODE was used to identify clusters (highly interconnected regions) in a network where clusters were often protein complexes and parts of pathways. Additionally, clusters in a protein similarity network represent protein families [66]. *P* < 0.05 were chosen as the significance threshold.

### RBPs-based prognostic model construction

Using the “survival” package in R and Univariate Cox regression analysis, we confirmed relevant prognostic signatures from differentially expressed RBPs. *P* < 0.05 was considered statistically significant. In total, 11 gene signatures were selected by Lasso Cox regression analysis using the “glmnet” package. Next, we calculated a risk score to assess patient prognoses. The risk score formula for each sample was calculated according to RBPs expression (*Expi*) and coefficient value (*βi*): risk score= exp (0.048* BRIX1+ 0.014* DYNC1H1+ 0.017* GTPBP4+ 0.002* PRKDC+ 0.00001* RAN+ 0.022* RBM19+ 0.001* SF3B4+ 0.006* SMG5+ 0.027* SPATS2+ 0.011*TAF9+ 0.023* THOC5). HCC patients were divided into low-risk group and high-risk group based on the median of risk score values. Then, we used the “survival” package to compare differences of OS between the two groups. Additionally, a receiver operating characteristic (ROC) curve analysis was implemented using the “survivalROC” package to evaluate the prognostic capability of this model.

### Correlation analysis of clinical traits

Boxplot illustrated by using "beeswarm" package was aimed to explore the relationship between the 11 RBPs and clinicopathologic features. *P* < 0.05 was considered statistically significant.

### Genomic analysis and drug-prognostic RBPs interaction

The cBioPortal for Cancer Genomics (https://www.cbioportal.org/) was an open access tool which allowed for analysis, visualization, and downloads of various cancer genomics datasets [67]. We compared the genomic alterations of the selected 11 RBPs in HCC (TCGA, Firehose Legacy). The DGIdb database (http://www.dgidb.org/) was searched for candidate drugs for HCC patients based on prognostic RBPs.

### Validation of the prognostic RBPs expression in the HPA database

The immunohistochemistry staining of the 11 prognostic RBPs was detected in the Human Protein Atlas database (HPA, https://www.proteinatlas.org/) [68].

### Clinical samples and immunohistochemistry staining

In total, 98 HCC patient tissues were collected from the Peking University Cancer Hospital and Institute. Clinical research was approved by the ethics committee of the Peking University Cancer Hospital and Institute. None of patients received any preoperative treatment. All tissues were histologically confirmed by pathologists. Immunohistochemistry staining was performed according to previously published protocols [69]. In brief, the staining index (0-12) was calculated by staining intensity (negative-0; weak-1; moderate-2; or strong-3) multiplying the percentage of SPATS2 positive staining (<5%-0; 5% ~ 25%-1; 25% ~ 50%-2; 50% ~ 75%-3;>75%-4). If the staining index of tumor tissues was greater than or equal to the median, then the expression of SPATS2 protein was identified as upregulated, and vice versa. The antibody used in this study was anti-SPATS2 (Bioss Inc, USA).

### Cell culture and transfection

HepG2 and Huh7 cells were cultured in Dulbecco’s Modified Eagle Medium (DMEM) with 10% fetal bovine serum and incubated in a humidified chamber in 5% CO_2_ at 37° C. Two synthesized siRNAs were transfected into cells with Lipofectamine 2000™ (Invitrogen) according to manufacturer’s instructions. Knockdown efficiency for SPATS2 in HCC cells was confirmed by western blot.

### Cell growth assay and EdU cell proliferation assay

In total, 2 × 10^3^ HCC cells were suspended per well and added into 96-well culture plates. After 24 h, the cells were incubated with CCK8 solution for 1 h and measured at OD450 using a microplate reader. In the EdU assay, a total of 5 × 10^4^ HCC cell suspension per well were seeded into 96-well culture plates. After 24 h, the EdU solution was added to label S phase cells and Apollo staining was conducted based on the instructions. Each assay was repeated in triplicate.

### Cell migration assays

For migration assay, 5 × 10^4^ cells suspended in serum-free DMEM were seeded into the internal of the transwell chamber (Corning, NY, USA), while outside the chamber contained DMEM with 10% fetal bovine serum. After 36 hours, chambers were fixed with 4% paraformaldehyde, stained with crystal violet, and counted under a microscope. The assay was repeated in triplicate.

### Statistical analyses

Statistical analyses were performed using the SPSS 20.0 software (SPSS, Inc., Chicago, IL). The overall and disease-free survival curves were plotted using Kaplan-Meier analysis, and differences were evaluated by log-rank test. The Pearson and chi-square test were used to examine the relationship between SPATS2 expression and clinicopathological features. Univariate and multivariate Cox analyses were used to assess independent prognostic factors. A Student’s t-test was performed to compare differences between two groups of samples. *P* < 0.05 was regarded as statistically significant.

## Supplementary Material

Supplementary Table 1

Supplementary Tables 2 and 3
