# Supplementary material for: Integrated bioinformatic analysis of RNA binding proteins in hepatocellular carcinoma
Source: Aging (Albany NY). 2020 Dec 19;13(2):2480–505. doi: 10.18632/aging.202281 (PMC7880356; doi:10.18632/aging.202281)
Supplement: Supplementary Table 1 [file aging-13-202281-s001.docx]

Supplementary Table 1. The function of module 1 within PPI network.

| Term ID | Term description | Observed gene count | Background gene count | False discovery rate | Matching proteins in your network (IDs) | Matching proteins in your network (labels) |
| --- | --- | --- | --- | --- | --- | --- |
| hsa03040 | Spliceosome | 35 | 130 | 5.93E-58 | ENSP00000218224, ENSP00000221494, ENSP00000243563, ENSP00000244520, ENSP00000252622, ENSP00000254193, ENSP00000266079, ENSP00000266735, ENSP00000269349, ENSP00000269601, ENSP00000271628, ENSP00000283179, ENSP00000300413, ENSP00000306614, ENSP00000307863, ENSP00000310448, ENSP00000312981, ENSP00000315379, ENSP00000324122, ENSP00000326806, ENSP00000341826, ENSP00000342374, ENSP00000359645, ENSP00000364813, ENSP00000365625, ENSP00000376309, ENSP00000392094, ENSP00000400591, ENSP00000412566, ENSP00000418563, ENSP00000421592, ENSP00000434359, ENSP00000463058, ENSP00000469468, ENSP00000472998 | ALYREF, DHX16, EFTUD2, EIF4A3, HNRNPA1, USP39, HNRNPA3, HNRNPU, LSM2, LSM4, LSM7, NCBP2, PPIH, PQBP1, PRPF3, PRPF31, PRPF6, PUF60, RBM8A, RBMX, SART1, SF3A2, SF3B4, SNRNP70, SNRPA, SNRPA1, SNRPB, SNRPC, SNRPD1, SNRPD2, SNRPE, SNRPF, TXNL4A, U2AF2, U2SURP, |
| hsa03015 | mRNA surveillance pathway | 13 | 89 | 1.91E-17 | ENSP00000238112, ENSP00000245934, ENSP00000264645, ENSP00000269349, ENSP00000276201, ENSP00000292476, ENSP00000326806, ENSP00000345412, ENSP00000362063, ENSP00000421592, ENSP00000457723, ENSP00000463058, ENSP00000484669 | ALYREF, CASC3, CPSF1, CPSF3, CPSF4, CPSF7, CSTF2, EIF4A3, NCBP2, RBM8A, RNPS1, SYMPK, UPF3B |
| hsa03020 | RNA polymerase | 6 | 31 | 5.90E-09 | ENSP00000292614, ENSP00000301788, ENSP00000324124, ENSP00000342889, ENSP00000403852, ENSP00000415536 | POLR2F, POLR2G, POLR2H, POLR2J, POLR2K, POLR2L |
| hsa00240 | Pyrimidine metabolism | 6 | 100 | 2.28E-06 | ENSP00000292614, ENSP00000301788, ENSP00000324124, ENSP00000342889, ENSP00000403852, ENSP00000415536 | POLR2F, POLR2G, POLR2H, POLR2J, POLR2K, POLR2L |
| hsa03013 | RNA transport | 7 | 159 | 2.28E-06 | ENSP00000264645, ENSP00000269349, ENSP00000276201, ENSP00000326806, ENSP00000421592, ENSP00000457723, ENSP00000463058 | ALYREF, CASC3, EIF4A3, NCBP2, RBM8A, RNPS1, UPF3B |
| hsa00230 | Purine metabolism | 6 | 173 | 3.86E-05 | ENSP00000292614, ENSP00000301788, ENSP00000324124, ENSP00000342889, ENSP00000403852, ENSP00000415536 | POLR2F, POLR2G, POLR2H, POLR2J, POLR2K, POLR2L |
| hsa05016 | Huntington's disease | 6 | 193 | 6.02E-05 | ENSP00000292614, ENSP00000301788, ENSP00000324124, ENSP00000342889, ENSP00000403852, ENSP00000415536 | POLR2F, POLR2G, POLR2H, POLR2J, POLR2K, POLR2L |
| hsa05169 | Epstein-Barr virus infection | 6 | 194 | 6.02E-05 | ENSP00000292614, ENSP00000301788, ENSP00000324124, ENSP00000342889, ENSP00000403852, ENSP00000415536 | POLR2F, POLR2G, POLR2H, POLR2J, POLR2K, POLR2L |
| hsa04623 | Cytosolic DNA-sensing pathway | 4 | 62 | 7.68E-05 | ENSP00000324124, ENSP00000342889, ENSP00000403852, ENSP00000415536 | POLR2F, POLR2H, POLR2K, POLR2L |
| hsa03018 | RNA degradation | 3 | 77 | 0.0027 | ENSP00000252622, ENSP00000364813, ENSP00000469468 | LSM2, LSM4, LSM7 |
| hsa05322 | Systemic lupus erythematosus | 2 | 94 | 0.0477 | ENSP00000300413, ENSP00000412566 | SNRPB, SNRPD1 |
