# Supplementary material for: Integrated bioinformatic analysis of RNA binding proteins in hepatocellular carcinoma
Source: Aging (Albany NY). 2020 Dec 19;13(2):2480–505. doi: 10.18632/aging.202281 (PMC7880356; doi:10.18632/aging.202281)
Supplement: Supplementary Tables 2 and 3 [file aging-13-202281-s002.pdf]

SUPPLEMENTARY TABLES

Supplementary Table 2. The function of module 2 within PPI network.

Term ID	Term description	Observed gene count	Background gene count	False discovery rate	Matching proteins in your network (IDs)	Matching proteins in your network (labels)
hsa03010	Ribosome	18	130	7.24E-22	ENSP00000222247, ENSP00000230050, ENSP00000262584, ENSP00000270625, ENSP00000272317, ENSP00000346015, ENSP00000346037, ENSP00000346080, ENSP00000357555, ENSP00000379506, ENSP00000400467, ENSP00000404375, ENSP00000416429, ENSP00000418082, ENSP00000428085, ENSP00000429374, ENSP00000472469, ENSP00000472985	RPL14, RPL18A, RPL22L1, RPL27A, RPL30, RPL32, RPL36A, RPL37A, RPL8, RPL9, RPLP1, RPS11, RPS12, RPS20, RPS27, RPS27A, RPS28, RPS5
hsa03013	RNA transport	19	159	7.24E-22	ENSP00000216190, ENSP00000220849, ENSP00000245838, ENSP00000253108, ENSP00000258742, ENSP00000261600, ENSP00000265097, ENSP00000313007, ENSP00000326531, ENSP00000354125, ENSP00000356448, ENSP00000364448, ENSP00000369391, ENSP00000379182, ENSP00000379475, ENSP00000389182, ENSP00000420306, ENSP00000429931, ENSP00000436679	DDX39B, EIF3B, EIF3D, EIF3E, EIF3G, EIF3H, NUPL2, NXF1, PABPC1, RAE1, RPP30, RPP40, THOC1, THOC2, THOC3, THOC5, THOC6, TPR, UPF3A
hsa03008	Ribosome biogenesis in eukaryotes	12	76	1.77E-15	ENSP00000221801, ENSP00000225298, ENSP00000261708, ENSP00000264279, ENSP00000321449, ENSP00000355541, ENSP00000369162, ENSP00000369391, ENSP00000370589, ENSP00000377944, ENSP00000389182, ENSP00000436679	FBL, HEATR1, NOP56, NOP58, NXF1, RIOK1, RPP30, RPP40, RRP7A, UTP14A, UTP18, UTP6
hsa03018	RNA degradation	8	77	2.82E-09	ENSP00000221233, ENSP00000258169, ENSP00000313007, ENSP00000315476, ENSP00000359939, ENSP00000361433, ENSP00000368984, ENSP00000374354	EXOSC1, EXOSC2, EXOSC4, EXOSC5, EXOSC8, EXOSC9, MPHOSPH6, PABPC1

hsa03015	mRNA surveillance pathway	6	89	3.44E-06	ENSP00000313007, ENSP00000355261, ENSP00000364448, ENSP00000379475, ENSP00000425133, ENSP00000436679	DDX39B, NXF1, PABPC1, SMG5, SMG7, UPF3A
hsa03040	Spliceosome	4	130	0.0031	ENSP00000245838, ENSP00000261600, ENSP00000265097, ENSP00000379475	DDX39B, THOC1, THOC2, THOC3
hsa05164	Influenza A	3	168	0.0444	ENSP00000379182, ENSP00000379475, ENSP00000436679	DDX39B, NXF1, RAE1

Supplementary Table 3. The function of module 3 within PPI network.

Term ID	Term description	Observed gene count	Background gene count	False discovery rate	Matching proteins in your network (IDs)	Matching proteins in your network (labels)
hsa03010	Ribosome	8	130	1.44E-14	ENSP00000288937, ENSP00000296102, ENSP00000306548, ENSP00000354525, ENSP00000354580, ENSP00000357823, ENSP00000384952, ENSP00000461930	MRPL13, MRPL17, MRPL21, MRPL24, MRPL33, MRPL9, MRPS12, MRPS21
